# Analysis of Upper Airway Morphology Using Four‐Dimensional Dynamic MRI With Active Deep Learning‐Based Automatic Segmentation

**DOI:** 10.1002/jmri.70237

**Published:** 2026-01-28

**Authors:** Cheng‐Yang Yu, Meng‐Chen Chung, Yunn‐Jy Chen, Han‐Wei Wang, Jonathan X. Zhou, Shih‐Lung Chen, Kevin T. Chen, Tiffany Ting‐Fang Shih

**Affiliations:** ^1^ Department of Biomedical Engineering National Taiwan University Taipei Taiwan; ^2^ Sleep Center National Taiwan University Hospital Taipei Taiwan; ^3^ Department of Dentistry National Taiwan University Hospital Taipei Taiwan; ^4^ Meinig School of Biomedical Engineering Cornell University Ithaca New York USA; ^5^ Department of Radiology College of Medicine, National Taiwan University Taipei Taiwan; ^6^ Department of Medical Imaging National Taiwan University Hospital Taipei Taiwan; ^7^ Shin Kong Wu Ho Su Memorial Hospital Taipei Taiwan

**Keywords:** active learning, deep learning, free‐breathing 4D MRI, image segmentation, open mouth breathing, upper airway

## Abstract

**Background:**

Upper‐airway morphology changes during breathing can be captured with cine 4D MRI. Active‐learning nnU‐Net reduces manual labeling while maintaining accuracy.

**Purpose:**

For automatic upper airway segmentation on free‐breathing cine 4D MRI using active learning and quantifying dynamic changes under two mouth positions.

**Study Type:**

Prospective cross‐sectional study.

**Population:**

Eighty‐four OSA (obstructive sleep apnea)‐free adults (28 M/56F; 18–80 years; 33 with sleep‐related breathing symptoms). Segmentation performance was evaluated on an internal test set (*n* = 18).

**Fieldstrength/Sequence:**

3T, free‐breathing time‐resolved imaging with interleaved stochastic trajectories (TWIST) sequence under closed‐ and open‐mouth positions.

**Assessment:**

Manual annotations by a technologist (radiologist‐verified) served as reference standard and training labels for an active‐learning nnU‐Net (68 training; four fixed validation). Total airway length, cross‐sectional area (CSA), and total airway volume were computed at each anatomical level and compared across mouth positions, sex, and sleep‐related symptom status, and independent predictors were identified.

**Statistical Tests:**

Paired/unpaired t or Mann–Whitney *U* test (two‐sided *p* = 0.05). Predictor selection by 10‐fold LASSO; effects estimated via ordinary least squares with cluster‐robust standard errors.

**Results:**

Segmentation achieved a dice 0.959 ± 0.019 (test set). Open‐mouth breathing significantly lengthened the total airway (7.92 ± 1.07 vs. 7.41 ± 0.93 cm) and reduced retropalatal CSA (1.51 ± 0.68 vs. 1.80 ± 0.69 cm^2^). Coefficients of variation (CVs) for CSA and volume were significantly higher with 20‐s open‐mouth breathing. Males (*n* = 28) exhibited significantly larger airway volumes than females (closed 27.94 ± 4.87 vs. 19.82 ± 3.26 cm^3^; open 30.26 ± 5.94 vs. 20.94 ± 3.85 cm^3^). Symptomatic individuals (*n* = 33) had significantly longer airways (closed 7.96 ± 0.96 vs. 7.04 ± 0.70 cm; open 8.54 ± 1.01 vs. 7.52 ± 0.91 cm), narrower open‐mouth retropalatal CSA (1.24 ± 0.51 vs. 1.68 ± 0.72 cm^2^), and greater retropalatal CSA dynamic variability. Multivariable regression confirmed mouth position, symptoms, and sex as independent predictors.

**Data Conclusion:**

Four‐dimensional cine MRI with active‐learning nnU‐Net can automatically quantify dynamic upper airway morphology, demonstrating systematic differences and dynamic variability.

**Evidence Level:**

2.

**Technical Efficacy:**

Stage 2.

## Plain Language Summary

This study investigates how open‐mouth breathing alters the shape and stability of the upper airway. Using advanced dynamic MRI and artificial intelligence, we automatically tracked airway movements in 84 adults without obstructive sleep apnea. We discovered that breathing with an open mouth significantly lengthens the airway and narrows the back of the throat, making it less stable. Individuals who snore showed similar structural changes to those with sleep apnea, suggesting these may be early warning signs. Our AI‐driven approach provides a non‐invasive way to uncover dynamic airway structures and understand respiratory health.

## 1 Introduction

The morphology and contour of the upper airway are important to physiological functions such as respiration, phonation, and deglutition. Mouth position, whether open or closed, markedly affects both airway structure and breathing physiology. An open mouth decreases the magnitude of negative critical closing pressure, thereby increasing the risk of airway collapse [1]. It also simultaneously lengthens and narrows the airway [2], further increasing its susceptibility to collapse. Moreover, multiple studies have identified open‐mouth breathing during sleep as a risk factor for obstructive sleep apnea (OSA) [3, 4, 5].

Magnetic resonance imaging (MRI) provides a noninvasive and comprehensive assessment of airway morphology and surrounding soft tissues [6, 7]. Previous studies have used either three‐dimensional (3D) static or two‐dimensional (2D) dynamic MRI sequences to characterize the structure of the airway [7, 8, 9]. While the 3D static sequence offers high spatial resolution and comprehensive volumetric coverage, it was unable to capture airway changes during natural tidal breathing [6, 7]. On the other hand, 2D dynamic MRI sequences can track airway changes throughout the respiratory cycle but are constrained to a single imaging plane [7]. To address these challenges, the time‐resolved Imaging with interleaved stochastic trajectories (TWIST) MRI sequence has been used for its ability to acquire 3D images with a temporal resolution of approximately one frame per second [10, 11, 12, 13], allowing four‐dimensional visualization of the airway's dynamic movement.

Since 4D MRI produces a continuous volumetric time‐series, manual annotation requires repetitive delineation of 3D structures. This renders the process prohibitively time‐consuming and susceptible to inter‐observer variability. To address these challenges, automated segmentation using deep learning has become essential. Specifically, the nnU‐Net framework has demonstrated outstanding performance in extracting intricate features across various medical imaging tasks [14, 15, 16, 17, 18], making it an ideal candidate for automating airway segmentation. Furthermore, to mitigate the substantial burden of manually labeling large training datasets, active learning strategies can be employed to selectively identify critical data points [19, 20, 21], thereby accelerating the training process. We anticipated that TWIST MRI acquired during normal breathing would provide deeper insights into dynamic respiratory physiology and the effects of mouth position (closed versus open) on airway structure and function. Therefore, this study aimed to comprehensively characterize dynamic upper‐airway morphology under these conditions using TWIST MRI in combination with an active‐learning nnU‐Net framework for automatic segmentation.

## 2 Materials and Methods

### 2.1 Study Participants

This prospective, observational, cross‐sectional study recruited 90 participants using the following inclusion criteria: (a) aged 18–80 years, (b) absence of major systemic diseases, (c) no history or diagnosis of obstructive sleep apnea (OSA), (d) no contraindications to MRI, and (e) not planning pregnancy. All MRI scans and sleep apnea clinical score (SACS) questionnaires were performed between December 2022 and October 2023. The study was sponsored by the Taiwan National Science and Technology Council (112‐2314‐B‐002‐245, 110‐2314‐B‐002‐143) and was approved by the institutional IRB. Written informed consent was obtained from all participants prior to enrollment and imaging. Participants who self‐reported snoring or other sleep‐related symptoms were classified as symptomatic and referred to the outpatient sleep clinic for further evaluation.

Those who received comprehensive examinations and were subsequently diagnosed with OSA were excluded from the final statistical analysis but still retained for segmentation model training.

### 2.2 MRI Measurements

All data used in the study were acquired with a 3.0 Tesla SIEMENS MAGNETOM Vida scanner (Erlangen, Germany) using a T1‐weighted 3D TWIST sequence. The key acquisition parameters were: TR/TE = 2.0/0.59 ms; flip angle = 10°; field of view = 240 × 240 mm^2^; and voxel size = 1.25 × 1.25 × 2.0 mm^3^. To achieve a temporal resolution of 0.99 s per volume, the TWIST k‐space sampling was configured with a central region (A) of 20% and a peripheral sampling density (B) of 20% using a symmetric share reconstruction scheme. Parallel imaging was performed using CAIPIRINHA with a total acceleration factor of 4 (2 × 2). Additional techniques included 7/8 partial Fourier in both phase and slice directions and a receiver bandwidth of 1160 Hz/Px.

For the four‐dimensional (4D) dynamic imaging of the airway, participants underwent pre‐imaging training to ensure adherence to the breathing protocol. During the first 20 s of imaging, the participants were instructed to maintain a closed‐mouth position and breathe naturally. For the next 20 s, the participants were asked to bite down on a 10‐mL syringe wrapped in gauze to simulate an open‐mouth position while continuing to breathe. This protocol resulted in 20 consecutive 3D image sets for both closed‐ and open‐mouth conditions. The syringe was wrapped in gauze to enhance participant comfort and was consistently positioned between the maxillary lateral incisor and the canine on each side. This specific location was selected because the natural anatomical prominence of the canine provides a stable anchor point, which effectively prevents the syringe from slipping and ensures a consistent bite posture throughout the scan duration.

A radiographic technologist (T.T.‐F.S., 31 years of experience) annotated the MR images manually for two regions of interest (ROI): (1) the airway ventral to the epiglottis (referred to as the “epiglottic airway”), and (2) the airway segment extending from 1 cm above the hard palate through 1 cm below the epiglottic base, with the region selected in (1) excluded. All segmentations were confirmed by a radiologist (C.H.C, with 35 years of experience). The epiglottis region was specifically selected for its potential influence on airway control during breathing.

### 2.3 Segmentation Model Development

The nnU‐Net framework was used for the segmentation task. A dataset‐specific “fingerprint,” computed from the input images, was used to identify and resample relevant regions. A 3D U‐Net architecture where the ReLU activation functions were replaced with leaky ReLUs (negative slope = 0.01) was then trained with the resampled data. The model took 3D MR images as input and generated 3D segmentation maps, assigning each voxel the class with the highest predicted probability: 0 = background, 1 = upper airway, and 2 = epiglottic airway. All trained networks were implemented using PyTorch 2.4.0 and an NVIDIA RTX 3070ti GPU with 8 GB memory. Hyperparameters, including the learning rate and weight decay, were automatically determined by the computed fingerprint. The loss function combined Dice and cross‐entropy losses in a 1:1 ratio, and model optimization used stochastic gradient descent with Nesterov momentum (*μ* = 0.99). Model performance was evaluated using the Dice coefficient.

To mitigate the burden of manual labeling, an active learning methodology was implemented such that just a limited amount of data for manual segmentation was required to produce training labels for the dataset (90 samples, 3600 3D images in total).

*Data partitioning*. The 90 samples in the dataset were evenly divided into five groups, where the median fingerprint values (median shape and intensity distribution) generated by the nnU‐Net did not differ significantly among the five groups. The data were assigned to specific roles for the active learning pipeline: (1) initial training set: 14 participants from Group 1 were fully manually labeled to serve as the seed data for the first model. (2) Fixed validation set: four participants from Group 1 were fully manually labeled and reserved strictly as a fixed validation set. This set was used to monitor convergence and prevent overfitting across all active learning generations. (3) Unlabeled candidate pool: Groups 2, 3, and 4 served as the reservoir of unlabeled data, introduced sequentially into the candidate pool across generations. (4) Hold‐out test set: Group 5 was fully labeled and held out completely until the final evaluation.

*Active Learning Strategy*. We employed an uncertainty‐based query strategy. For each image evaluated by the current model, voxel‐wise uncertainty was quantified using entropy

$$\text{Entropy}=-(p\left(\mathrm{BG}\right)\log\left(p\left(\mathrm{BG}\right)\right)+p\left(\mathrm{AW}\right)\log\left(p\left(\mathrm{AW}\right)\right)+p\left(\mathrm{EP}\right)\log\left(p\left(\mathrm{EP}\right)\right)$$

where $p\left(\mathrm{BG}\right)$, $p\left(\mathrm{AW}\right)$, and $p\left(\mathrm{EP}\right)$ represent the predicted probabilities for the background, upper airway, and epiglottic airway, respectively. The median of the voxel‐wise entropy values was utilized as the representative uncertainty score for each 3D image. To establish the selection threshold, we calculated the population mean and standard deviation (SD) of these scores across the candidate pool. Any image whose score exceeded this population mean by more than one SD was classified as high‐entropy.

*Iterative training workflow*. The model evolved through four generations. In each iteration, the candidate pool was progressively expanded, and the model assessed the uncertainty of all images within this pool, regardless of their prior inclusion in the training set. The fixed validation set was strictly excluded from this pool and used solely for performance monitoring of iterative training. Generation 1: the Gen‐1 model was trained using the initial training set. Generation 2: the candidate pool was formed by combining the initial training set and Group 2. The Gen‐1 model assessed all images within the combined pool, and high‐entropy samples were identified. If the sample was new, it was manually labeled and added to the training set; if it was previously labeled and included in the training set but remained high‐entropy, it was re‐selected. This resulted in multiple inclusions of the same data point within the training set, thereby increasing the model's focus on these persistent hard examples. The Gen‐2 model was then retrained on this augmented dataset, which comprised the Initial Training Set plus the high‐entropy samples identified in this round. Generation 3: the candidate pool was expanded to include Group 3. The Gen‐2 model screened the cumulative pool (initial training set + Groups 2 and 3), adding both new high‐entropy samples and re‐selecting persistent hard examples from previous groups into the augmented dataset. The Gen‐3 model was then trained on this further expanded dataset. Generation 4: the candidate pool was fully expanded to include Group 4. The Gen‐3 model scanned the final cumulative pool (initial training set + Groups 2–4) to identify any remaining high‐uncertainty cases for the final training iteration. The Gen‐4 model was trained on this final cumulative dataset.

### 2.4 Image Analysis

Upper‐airway structures were automatically segmented in all volumetric MR frames using our final model (Gen‐4 model). All subsequent morphological measurements and statistical analyses were performed exclusively on these automated segmentation masks. For each participant and for each mouth position (closed/open), we analyzed 20 consecutive 3D frames. In every frame we quantified: (i) total airway length, defined as the distance from the soft palate to the epiglottic base; (ii) epiglottic airway length, defined as the distance from the epiglottic tip to the epiglottic base; (iii) airway volume, computed as the number of voxels labeled as airway multiplied by the voxel volume; and (iv) cross‐sectional area (CSA) at the retropalatal (RP) and retroglossal (RG) levels, as well as the frame‐wise mean CSA of the total airway. Specifically, the CSA for the RP and RG levels was defined as the minimum CSA identified within the retropalatal space and retroglossal space, respectively. For each metric, participant‐level summaries were obtained by averaging values across the 20 frames and reporting temporal variability as the coefficient of variation (CV = SD/mean).

### 2.5 Multivariate Linear Regression Analysis of the Airway Measurements

For each airway metric (airway length, CSA, and volume), we constructed a separate multivariate linear regression model to identify independent predictors. Initially, the same set of eight candidate predictors was considered for all models: mouth position (mouth open vs. closed during imaging), sex, and symptomatic status (binary variables), age, neck circumference, body mass index (BMI), height, and weight (continuous variables). All continuous variables and each airway metric were *Z*‐standardized (mean = 0, SD = 1) prior to modeling to yield standardized regression coefficients that are comparable in magnitude. We employed the least absolute shrinkage and selection operator (LASSO) regression with 10‐fold cross‐validation to select a parsimonious subset of predictors for each outcome. This penalized regression method shrinks less important coefficients to zero; variables with non‐zero coefficients are selected as key variables.

Using the predictors selected by LASSO, we refit each final model with ordinary least squares. Because each participant contributed two correlated measurements (mouth closed and mouth open), we treated each participant as a separate cluster and calculated cluster‐robust standard errors [22] to account for the resulting within‐person residual correlation.

### 2.6 Statistical Analysis

All statistical analyses in this study were conducted with Python 3.10.12 and SciPy 1.11.4.

For the active learning process, comparisons of segmentation uncertainty (entropy) across the four model generations were performed using the Kruskal–Wallis test, followed by Dunn's post hoc test [23]. *p*‐values for these multiple pairwise comparisons were adjusted using the Bonferroni correction [23].

For clinical airway measurements, data normality was assessed with the Shapiro–Wilk test and homogeneity of variance with Levene's test. Normally distributed data with equal variances were compared using the student's *t*‐test; those with unequal variances used the Welch's *t*‐test. Non‐normally distributed data were analyzed with the Mann–Whitney *U* test. Categorical variables were evaluated using Fisher's exact test. A two‐sided *p* < 0.05 denoted statistical significance. Clinical comparisons included (i) paired comparisons between closed and open mouth scans, (ii) independent comparisons between males and females, and (iii) independent comparisons between symptomatic and asymptomatic participants. For prespecified contrasts that were not statistically significant at two‐sided *p* ≥ 0.05, we reported 95% confidence interval. Parametric data were summarized as mean differences with *t*‐based 95% confidence intervals. Non‐parametric data were summarized as median differences with percentile bootstrap 95% confidence intervals based on 10,000 resamples, using paired resampling by subject for open–closed mouth and stratified resampling within groups for independent comparisons. Given the physiological interdependence of the airway metrics (e.g., volume and length), *p*‐values for these morphological comparisons were reported without multiple comparison correction to avoid Type II errors, while exact *p*‐values are provided for interpretation.

To assess whether variations in mouth opening influenced airway metrics, Pearson correlation or Spearman rank correlation was performed between the extent of mouth opening (Δ*d*) and the clinical airway measurements. Detailed methodology regarding this analysis is described in the Supplementary material S4.

For the multivariate linear regression analysis, data distributions were adjusted prior to modeling to satisfy linear model assumptions. We addressed outliers by minorizing the continuous outcome values at the 1st and 99th percentiles. For airway variability measures expressed as CV, the outcome distributions were right‐skewed; therefore, we applied a logarithmic transformation to these CV outcomes to better approximate normality. Additionally, all continuous variables and each airway metric were *Z*‐standardized (mean = 0, SD = 1) to yield standardized regression coefficients that are comparable in magnitude. Finally, the validity of the linear model's assumptions was assessed using normal Q–Q plots, residual scatterplots, and variance inflation factors.

## 3 Results

### 3.1 Study Participants

**FIGURE 1:**
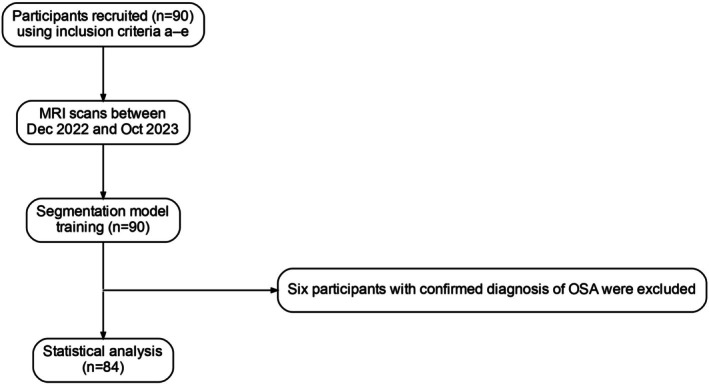
Flowchart of participant screening, inclusion, and exclusion. Inclusion criteria (a) aged 18–80 years; (b) no major systemic diseases; (c) no obstructive sleep apnea; (d) no MRI contraindications; and (e) no plans for pregnancy. Abbreviation: OSA, Obstructive sleep apnea.

A total of 90 participants were initially recruited. Following the MR study, six participants were diagnosed with OSA after referral for comprehensive examination and were consequently excluded from the statistical analysis. The final study cohort comprised 84 OSA‐free participants (mean age 41.27 ± 15.25 years; 28 men, 56 women), consisting of 33 symptomatic and 51 asymptomatic individuals. A flow diagram of recruitment and exclusion is shown in Figure 1.

The demographic and sleep‐related characteristics stratified by sex are summarized in Table 1, and those stratified by symptomatic status are summarized in Table 2. Male participants had significantly higher body weight, height, neck circumference, and SACS scores than females and a higher prevalence of snoring and gasping or choking during sleep. Those reporting either snoring or episodes of gasping or choking during sleep were deemed symptomatic; all others were asymptomatic. Compared with the asymptomatic group, symptomatic participants had a significantly higher mean age, body weight, BMI, neck circumference, and SACS score (5.70 ± 6.69 vs. 0.61 ± 0.72), while height remained comparable between groups (*p* = 0.326).

**TABLE 1:** Demographic and sleep‐related characteristics of the study cohort (*n* = 84), stratified by sex.

Characteristic	Overall	Male	Female	*p*
*N*	84	28	56	
Age (years)	41.27 ± 15.25 (23.00, 54.00)	39.18 ± 17.52 (23.00, 57.00)	42.32 ± 14.02 (36.00, 53.00)	0.638
BMI	23.55 ± 3.68 (21.26, 25.47)	23.70 ± 3.37 (22.04, 25.47)	23.48 ± 3.85 (21.23, 24.97)	0.592
Weight (kg)	62.98 ± 11.61 (55.00, 70.00)	70.36 ± 9.98 (64.00, 75.60)	59.29 ± 10.63 (53.00, 64.00)	< 0.001
Height (cm)	163.33 ± 8.48 (157.00, 170.00)	172.38 ± 4.89 (169.00, 175.00)	158.81 ± 5.86 (155.00, 163.00)	< 0.001
Neck circumference (cm)	34.21 ± 3.42 (32.00, 36.50)	36.79 ± 3.13 (35.50, 38.50)	32.93 ± 2.79 (31.00, 34.50)	< 0.001
SACs score	2.61 ± 4.88 (0.00, 2.00)	5.46 ± 7.30 (1.00, 5.00)	1.18 ± 1.89 (0.00, 1.00)	< 0.001
Snore condition (*n*)				0.047
Never	51	13	38	
Occasionally	16	5	11	
Consistently	17	10	7	
Gasping or choking during sleep (*n*)				0.005
Never	74	20	54	
Occasionally	6	5	1	
Consistently	4	3	1	

*Note*: Continuous variables are presented as mean ± standard deviation (with 25th and 75th percentiles); categorical variables are shown as counts (*n*). Body mass index (BMI) is calculated as weight (kg) divided by height (m)^2^. SACS = sleep apnea clinical score. *p*‐values compare males versus females.

**TABLE 2:** Demographic and sleep‐related characteristics of the study cohort (*n* = 84), stratified by symptomatic status.

Characteristic	Overall	Asymptomatic	Symptomatic	*p*
*N*	84	51	33	
Age (years)	41.27 ± 15.25 (23.00, 54.00)	35.14 ± 14.04 (22.00, 47.00)	50.76 ± 11.92 (46.00, 58.00)	< 0.001
BMI	23.55 ± 3.68 (21.26, 25.47)	22.72 ± 3.30 (20.78, 24.69)	24.84 ± 3.91 (22.58, 25.95)	0.010
Weight (kg)	62.98 ± 11.61 (55.00, 70.00)	60.22 ± 10.88 (51.00, 66.00)	67.23 ± 11.58 (60.00, 75.00)	0.006
Height (cm)	163.33 ± 8.48 (157.00, 170.00)	162.60 ± 8.96 (155.00, 169.00)	164.47 ± 7.66 (158.00, 170.00)	0.326
Neck circumference (cm)	34.21 ± 3.42 (32.00, 36.50)	32.97 ± 3.10 (30.50, 35.50)	36.14 ± 3.00 (33.50, 38.00)	< 0.001
SACs score	2.61 ± 4.88 (0.00, 2.00)	0.61 ± 0.72 (0.00, 1.00)	5.70 ± 6.69 (2.00, 7.00)	< 0.001
Snore condition (*n*)				< 0.001
Never	51	51	0	
Occasionally	16	0	16	
Consistently	17	0	17	
Gasping or choking during sleep (*n*)				< 0.001
Never	74	51	23	
Occasionally	6	0	6	
Consistently	4	0	4	

*Note*: “Symptomatic” refers to participants reporting sleep‐related symptoms; all participants were free of OSA. Continuous variables are presented as mean ± SD (25th, 75th percentiles); categorical variables are shown as counts (*n*). BMI and SACS are defined as in Table 1. *p*‐values compare asymptomatic versus symptomatic groups.

### 3.2 Automatic Segmentation and Active Learning: Dice Coefficient and Entropy Across Four Generations

**FIGURE 2:**
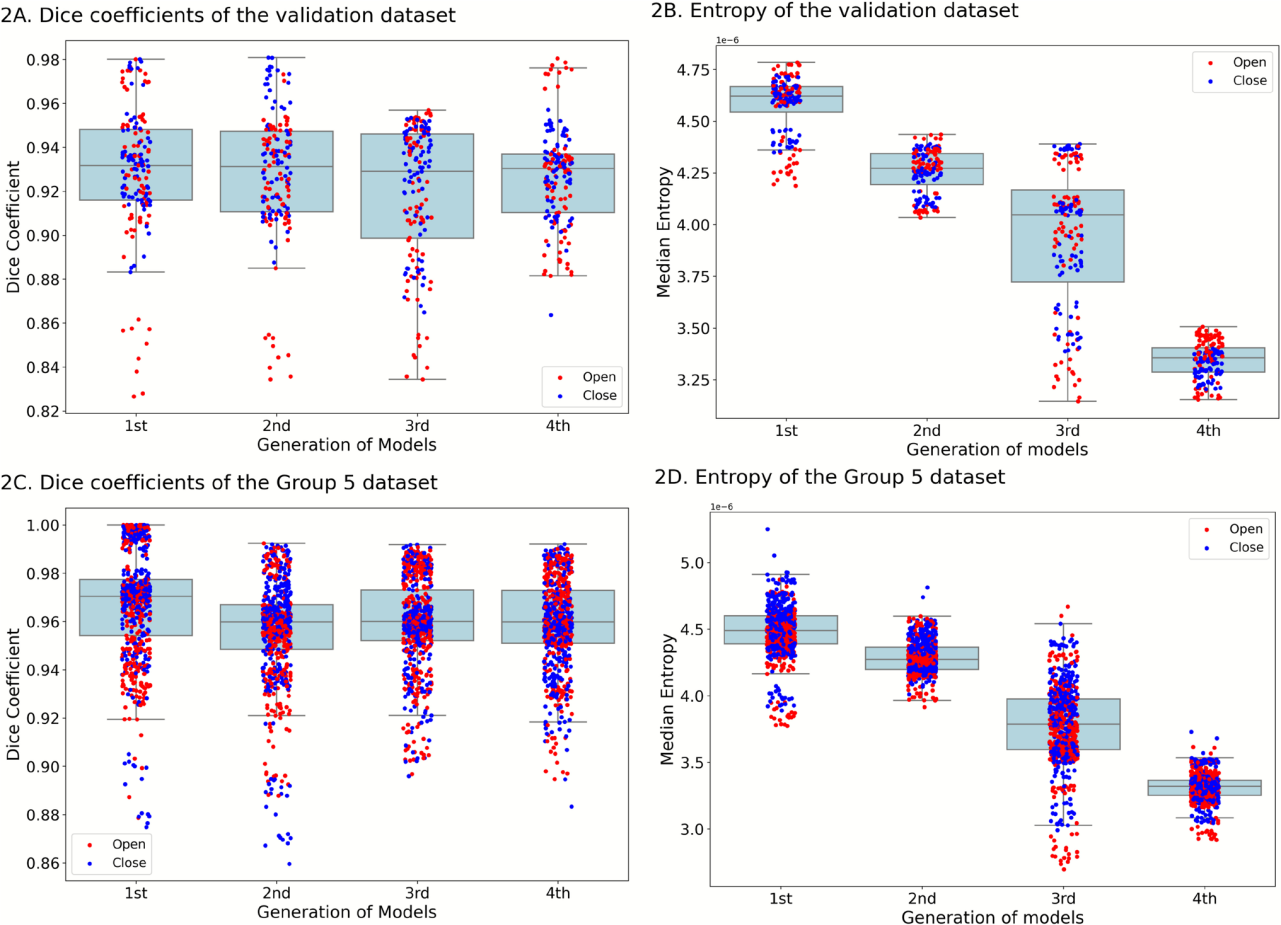
Dice coefficient and entropy across four model generations in the validation (A, B) and hold‐out test (C, D) datasets. Boxes indicate the interquartile range with medians, whiskers extend to the most extreme data points within 1.5 × IQR. Each dot denotes a single 3‐D image, with red indicating an open‐mouth scan and blue a closed‐mouth scan.

A total of 720, 1000, 1241, and 1701 images were used to train and progressively refine the four generations of models. Specifically, these datasets comprised 720, 834, 1016, and 1257 unique images for each respective generation, selected from a grand total of 2880 images. The manually labeled subset is significantly smaller than the full dataset. All models were validated using the same dataset of 160 labeled points drawn from four participants, and the Dice coefficient and Entropy distribution of the validation data are shown in Figure 2A,B.

**FIGURE 3:**
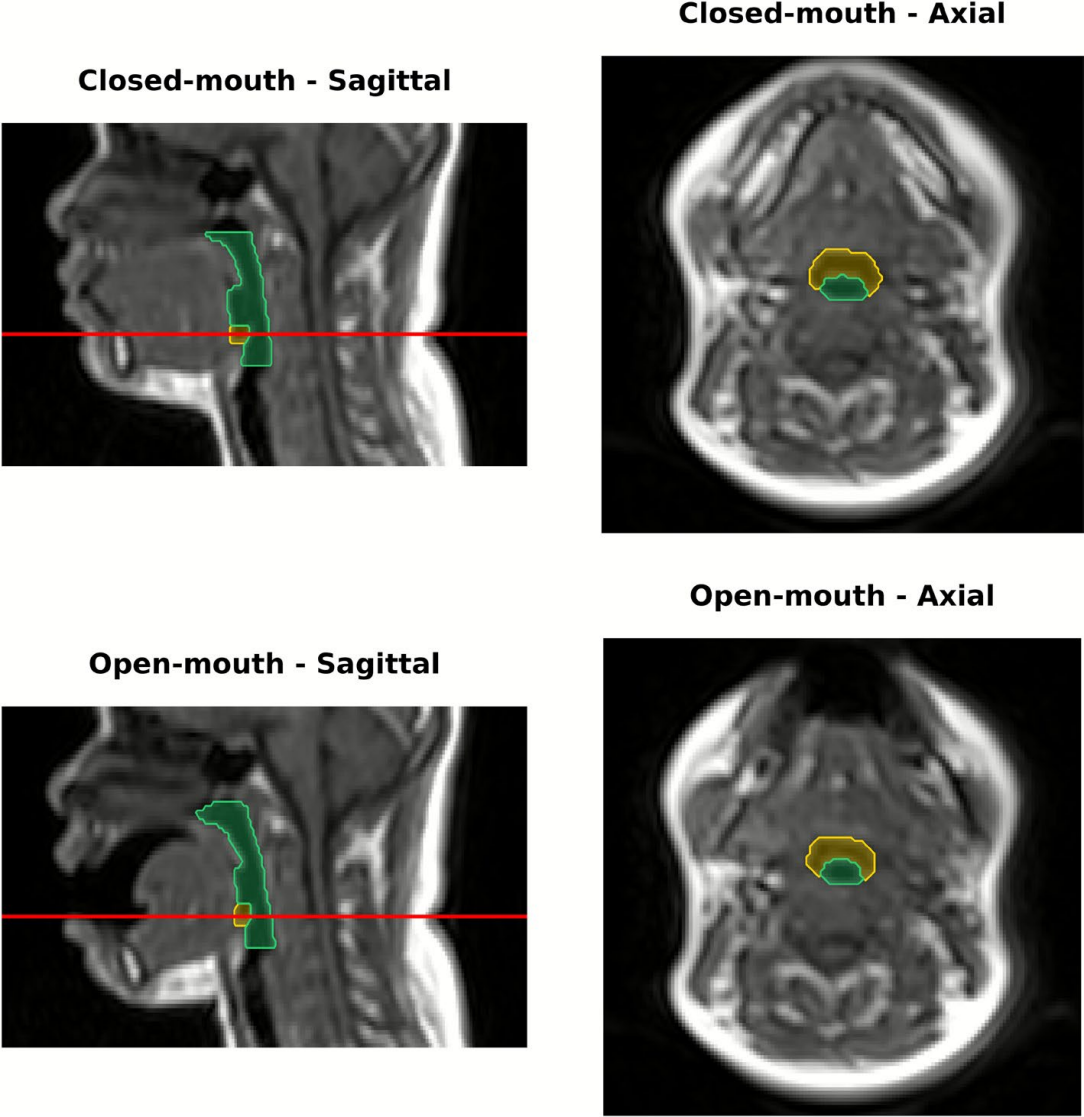
Representative sagittal (left) and axial (right) MR images of the upper airway in the closed (top) and open (bottom) mouth states with automated segmentations from the fourth‐generation model. The airway from 1 cm above the hard palate to 1 cm below the epiglottic base is overlaid in yellow, while the epiglottic airway alone is highlighted in green. On sagittal images, the red horizontal line indicates the axial slice level shown in the corresponding panels.

The dice coefficient and entropy distribution of the fully‐labeled hold‐out test set images (720 from 18 patients) are presented in Figure 2C,D. The segmentation examples of the 4th generation are shown in Figure 3. A summary of the Dice coefficient and entropy metrics for both the validation dataset and the hold‐out test dataset is provided in Table 3.

**TABLE 3:** Summary of the dice coefficient and entropy metrics across model generations for the validation group and hold‐out test dataset.

Model generation	Number of training images	Number of unique images	Validation dice coefficient	Validation entropy (e‐6)	Test dice coefficient	Test entropy (e‐6)
1	560	560	0.929 ± 0.030 (0.827–0.980)	4.57 ± 0.14 (4.19–4.78)	0.967 ± 0.023 (0.875–0.999)	4.48 ± 0.20 (3.77–5.25)
2	840	674	0.928 ± 0.029 (0.834–0.981)	4.26 ± 0.10 (4.03–4.44)	0.956 ± 0.022 (0.860–0.992)	4.29 ± 0.12 (3.91–4.81)
3	1081	856	0.919 ± 0.031 (0.834–0.957)	3.93 ± 0.35 (3.15–4.39)	0.959 ± 0.020 (0.896–0.992)	3.77 ± 0.32 (2.70–4.67)
4	1541	1097	0.927 ± 0.022 (0.864–0.981)	3.34 ± 0.09 (3.15–3.51)	0.959 ± 0.019 (0.883–0.992)	3.31 ± 0.11 (2.92–3.73)

*Note*: Results are given for the validation set (four subjects, 160 images) and for a hold‐out test set (18 subjects, 720 images). Values are presented as mean ± SD (range). Dice coefficient measures segmentation overlap; entropy quantifies output uncertainty. Entropy values are scaled by 10^−6^.

The results demonstrated a significant decrease in entropy across the four generations in both the validation and hold‐out test datasets. In contrast, the dice coefficients remain stable across generations within either dataset (validation range: 0.919–0.929; hold‐out test set range: 0.956–0.967). For the fourth‐generation model, the dice coefficient was 0.927 ± 0.022 on the validation set and 0.959 ± 0.019 on the Group 5 set. Dunn's post hoc test further verified that all pairwise entropy reductions were significant (Bonferroni‐adjusted).

### 3.3 General Features of Airway Morphology: Comparisons Between Closed and Open‐Mouth Position

The comparisons of airway measurements between closed‐ and open‐mouth positions are summarized in Table 4.

**TABLE 4:** Comparison of upper airway metrics between closed‐ and open‐mouth positions (*n* = 84).

Feature		Overall (*n* = 84)		
		Closed‐mouth	Open‐mouth	*p*
*Twenty‐second means*				
Length (cm)	Total airway	7.41 ± 0.93 (6.74, 7.83)*	7.92 ± 1.07 (7.17, 8.62)	< 0.001
	Epiglottic airway	1.28 ± 0.24 (1.13, 1.40)	1.29 ± 0.23 (1.18, 1.43)	0.298
CSA (cm^2^)	Average	3.03 ± 0.53 (2.66, 3.30)	3.02 ± 0.57 (2.62, 3.36)	0.821
	Retropalatal	1.80 ± 0.69 (1.34, 2.21)*	1.51 ± 0.68 (1.06, 1.77)	< 0.001
	Retroglossal	3.62 ± 0.85 (3.21, 4.06)	3.56 ± 1.05 (2.91, 3.89)	0.524
Volume (cm^3^)	Total airway	22.53 ± 5.44 (18.73, 25.68)*	24.04 ± 6.39 (19.03, 27.70)	< 0.001
	Epiglottic airway	2.86 ± 1.13 (2.16, 3.55)	2.88 ± 1.18 (2.23, 3.41)	0.839
*Twenty‐second coefficients of variation*				
CSA	Average	0.03 ± 0.02 (0.01, 0.03)*	0.04 ± 0.03 (0.02, 0.05)	< 0.001
	Retropalatal	0.06 ± 0.05 (0.04, 0.09)*	0.12 ± 0.12 (0.05, 0.15)	< 0.001
	Retroglossal	0.04 ± 0.02 (0.02, 0.05)*	0.07 ± 0.04 (0.03, 0.08)	< 0.001
Volume	Total airway	0.03 ± 0.02 (0.01, 0.03)*	0.04 ± 0.03 (0.02, 0.05)	< 0.001
	Epiglottic airway	0.06 ± 0.06 (0.03, 0.07)*	0.08 ± 0.07 (0.04, 0.09)	0.040

*Note*: Airway measurements include airway length (total airway: from soft palate to epiglottis base; epiglottic airway: from epiglottis tip to base), cross‐sectional areas (CSA) at the retropalatal and retroglossal levels, and volumes (total airway and epiglottic airway). Epiglottic airway is defined as the airway ventral to the epiglottis. For each metric, the mean and coefficient of variation (CV = SD/mean) over a 20‐s free‐breathing scan are shown as mean ± SD (25th, 75th percentiles). *p < 0.05 indicates a significant difference between closed and open mouth (paired test).

Among the entire study population (*n* = 84), the open‐mouth position was associated with a significantly longer total airway and larger total airway volume compared to the closed‐mouth position. Conversely, the retropalatal CSA was significantly smaller when the mouth was open. No significant differences were observed in the average CSA (*p* = 0.821), retroglossal CSA (*p* = 0.524), epiglottic airway length (*p* = 0.298), or epiglottic airway volume (*p* = 0.839). Regarding the 20‐s CV, the open‐mouth status exhibited significantly higher CV across all measured airway volumes and cross‐sectional areas. Exact *p*‐values and 95% confidence intervals for nonsignificant open–closed contrasts are summarized in Table S1.

The variability in Δ*d* achieved with the 10‐mL syringe was also assessed, showing a mean value of 16.77 ± 3.73 mm. Correlation analysis revealed no significant association between Δ*d* and any airway metrics; the detailed *p*‐value and confidence interval are summarized in Supplementary material S4.

### 3.4 Comparison of Airway Morphology Between Male and Female

The comparisons of the airway measurements between male and female participants are summarized in Table 5.

**TABLE 5:** Upper airway metrics under closed‐ and open‐mouth positions stratified by sex (male, *n* = 28; female, *n* = 56).

Feature		Closed‐mouth			Open‐mouth		
		Male (*n* = 28)	Female (*n* = 56)	*p*	Male (*n* = 28)	Female (*n* = 56)	*p*
*Twenty‐second means*							
Length (cm)	Total airway	8.31 ± 0.80 (7.60, 8.73)*	6.95 ± 0.60 (6.50, 7.44)	< 0.001	8.96 ± 0.85 (8.39, 9.50)*	7.40 ± 0.73 (6.87, 7.90)	< 0.001
	Epiglottic airway	1.51 ± 0.17 (1.40, 1.61)*	1.16 ± 0.17 (1.02, 1.30)	< 0.001	1.53 ± 0.18 (1.42, 1.57)*	1.18 ± 0.15 (1.15, 1.26)	< 0.001
CSA (cm^2^)	Average	3.36 ± 0.50 (3.00, 3.80)*	2.86 ± 0.46 (2.59, 3.22)	< 0.001	3.38 ± 0.60 (2.97, 3.72)*	2.84 ± 0.46 (2.47, 3.12)	< 0.001
	Retropalatal	1.94 ± 0.71 (1.47, 2.28)	1.74 ± 0.68 (1.32, 2.13)	0.225	1.64 ± 0.66 (1.09, 2.15)	1.44 ± 0.68 (1.04, 1.67)	0.206
	Retroglossal	4.12 ± 0.67 (3.73, 4.45)*	3.38 ± 0.82 (3.08, 3.92)	< 0.001	4.19 ± 1.31 (3.33, 4.73)*	3.25 ± 0.72 (2.80, 3.65)	0.001
Volume (cm^3^)	Total airway	27.94 ± 4.87 (25.21, 31.14)*	19.82 ± 3.26 (18.04, 21.74)	< 0.001	30.26 ± 5.94 (27.55, 32.72)*	20.94 ± 3.85 (18.15, 23.10)	< 0.001
	Epiglottic airway	3.92 ± 0.92 (3.41, 4.73)*	2.33 ± 0.81 (1.78, 2.83)	< 0.001	3.98 ± 1.12 (3.21, 4.81)*	2.32 ± 0.73 (1.96, 2.79)	< 0.001
*Twenty‐second coefficients of variation*							
CSA	Average	0.03 ± 0.02 (0.02, 0.03)	0.02 ± 0.01 (0.01, 0.03)	0.317	0.05 ± 0.03 (0.02, 0.06)	0.04 ± 0.03 (0.02, 0.05)	0.189
	Retropalatal	0.08 ± 0.06 (0.04, 0.09)	0.06 ± 0.03 (0.03, 0.08)	0.170	0.14 ± 0.13 (0.05, 0.18)	0.11 ± 0.11 (0.05, 0.12)	0.145
	Retroglossal	0.04 ± 0.03 (0.03, 0.06)	0.04 ± 0.02 (0.02, 0.04)	0.290	0.08 ± 0.06 (0.03, 0.09)	0.06 ± 0.03 (0.03, 0.08)	0.303
Volume	Total airway	0.03 ± 0.02 (0.02, 0.03)	0.02 ± 0.01 (0.01, 0.03)	0.317	0.05 ± 0.03 (0.02, 0.06)	0.04 ± 0.03 (0.02, 0.05)	0.189
	Epiglottic airway	0.06 ± 0.06 (0.03, 0.06)	0.06 ± 0.06 (0.03, 0.07)	0.673	0.08 ± 0.07 (0.04, 0.08)	0.08 ± 0.07 (0.04, 0.09)	0.462

*Note*: Metrics are defined as in Table 4 and are shown separately for males (*n* = 28) and females (*n* = 56) under closed and open mouth positions. Values are presented as mean ± SD (25th–75th percentiles). *p < 0.05 indicates a significant difference between sexes under the same mouth position (unpaired test).

Across both closed‐ and open‐mouth positions, male participants exhibited significantly larger airway dimensions. Specifically, total airway length, epiglottic airway length, average CSA, retroglossal CSA, total airway volume, and epiglottic airway volume were all significantly greater in males compared to females. In contrast, the retropalatal CSA did not differ significantly between sexes (closed‐mouth *p* = 0.225, open‐mouth *p* = 0.206). Furthermore, no significant sex‐related differences were observed in the 20‐s CV for any airway metric. Exact *p*‐values and 95% confidence intervals for nonsignificant sex comparisons are summarized in Table S2.

### 3.5 Comparison of Airway Morphology Between Symptomatic and Asymptomatic Individuals

The comparisons of the airway measurements between asymptomatic and symptomatic participants are summarized in Table 6.

**TABLE 6:** Upper airway metrics under closed‐ and open‐mouth positions, stratified by symptom status (asymptomatic, *n* = 51; symptomatic, *n* = 33).

Feature		Closed‐mouth			Open‐mouth		
		Asymptomatic (*n* = 51)	Symptomatic (*n* = 33)	*p*	Asymptomatic (*n* = 51)	Symptomatic (*n* = 33)	*p*
*Twenty‐second means*							
Length (cm)	Total airway	7.04 ± 0.70 (6.58, 7.52)*	7.96 ± 0.96 (7.38, 8.52)	< 0.001	7.52 ± 0.91 (6.83, 7.99)*	8.54 ± 1.01 (7.93, 9.18)	< 0.001
	Epiglottic airway	1.23 ± 0.24 (1.04, 1.40)*	1.35 ± 0.22 (1.20, 1.47)	0.003	1.24 ± 0.21 (1.15, 1.40)*	1.39 ± 0.23 (1.22, 1.50)	0.025
CSA (cm^2^)	Average	3.04 ± 0.57 (2.66, 3.37)	3.00 ± 0.47 (2.63, 3.24)	0.721	2.97 ± 0.55 (2.49, 3.27)	3.09 ± 0.61 (2.66, 3.53)	0.374
	Retropalatal	1.91 ± 0.67 (1.47, 2.22)	1.63 ± 0.70 (1.03, 2.00)	0.067	1.68 ± 0.72 (1.19, 1.93)*	1.24 ± 0.51 (0.95, 1.41)	0.002
	Retroglossal	3.56 ± 0.89 (3.20, 4.01)	3.72 ± 0.78 (3.27, 4.44)	0.390	3.35 ± 0.73 (2.81, 3.77)*	3.89 ± 1.36 (3.00, 4.65)	0.044
Volume (cm^3^)	Total airway	21.52 ± 5.09 (18.17, 23.87)*	24.08 ± 5.67 (19.81, 28.09)	0.040	22.41 ± 5.37 (17.85, 24.93)*	26.57 ± 7.07 (21.07, 32.17)	0.006
	Epiglottic airway	2.67 ± 1.05 (1.84, 3.33)	3.15 ± 1.20 (2.40, 4.11)	0.068	2.58 ± 0.98 (1.96, 3.17)*	3.33 ± 1.31 (2.48, 3.77)	0.007
*Twenty‐second coefficients of variation*							
CSA	Average	0.02 ± 0.01 (0.01, 0.03)	0.03 ± 0.02 (0.02, 0.04)	0.175	0.04 ± 0.03 (0.02, 0.05)	0.05 ± 0.03 (0.02, 0.06)	0.172
	Retropalatal	0.05 ± 0.03 (0.03, 0.06)*	0.09 ± 0.06 (0.04, 0.10)	< 0.001	0.09 ± 0.09 (0.04, 0.12)*	0.15 ± 0.15 (0.08, 0.16)	0.005
	Retroglossal	0.04 ± 0.02 (0.02, 0.04)	0.05 ± 0.03 (0.03, 0.06)	0.314	0.06 ± 0.03 (0.03, 0.07)	0.08 ± 0.05 (0.04, 0.09)	0.126
Volume	Total airway	0.02 ± 0.01 (0.01, 0.03)	0.03 ± 0.02 (0.02, 0.04)	0.175	0.04 ± 0.03 (0.02, 0.05)	0.05 ± 0.03 (0.02, 0.06)	0.172
	Epiglottic airway	0.06 ± 0.05 (0.03, 0.07)	0.06 ± 0.06 (0.03, 0.06)	0.869	0.08 ± 0.07 (0.04, 0.09)	0.08 ± 0.07 (0.04, 0.09)	0.783

*Note*: Metrics are defined as in Table 4 and are shown for asymptomatic (*n* = 51) and symptomatic (*n* = 33) participants under closed and open mouth positions. “Symptomatic” refers to individuals reporting sleep‐related symptoms; all participants were free of OSA. Values are presented as mean ± SD (25th, 75th percentiles). *p < 0.05 indicates a significant difference between asymptomatic and symptomatic groups under the same mouth position (unpaired test).

Regardless of mouth position, symptomatic individuals demonstrated significantly greater total airway length, epiglottic airway length, and total airway volume compared to asymptomatic individuals. Specific to the open‐mouth position, symptomatic participants exhibited significantly larger retroglossal CSA and epiglottic airway volume, whereas the retropalatal CSA was significantly smaller. No significant group differences were observed in average CSA (closed *p* = 0.721, open *p* = 0.374), closed‐mouth retroglossal CSA (*p* = 0.390), closed‐mouth retropalatal CSA (*p* = 0.067), or closed‐mouth epiglottic airway volume (*p* = 0.068). Regarding the 20‐s CV, symptomatic individuals exhibited significantly higher CV in the retropalatal CSA, regardless of mouth status. No significant group differences were found in the CVs of other metrics. Exact *p*‐values and 95% confidence intervals for nonsignificant symptomatic‐status comparisons are summarized in Table S3.

### 3.6 Multivariate Linear Regression Analysis of the Airway Measurements

**FIGURE 4:**
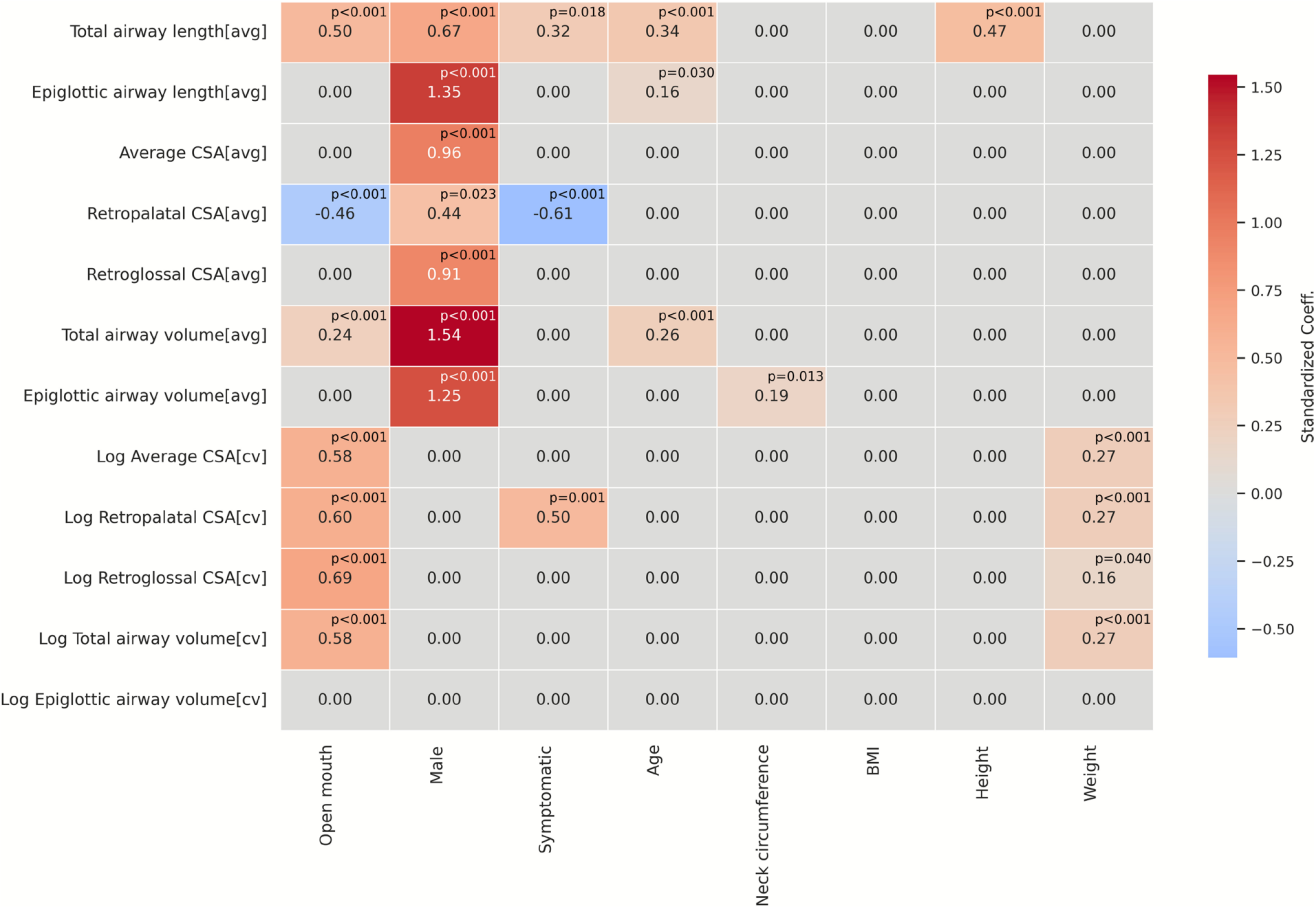
Heat‐map of standardized regression coefficients (*p*‐values annotated) for twelve upper‐airway metrics. Each row represents a multivariate linear model. Predictors were selected via LASSO regression with 10‐fold cross‐validation from eight candidate predictors: mouth status (open/closed), sex, symptom status, age, neck circumference, body‐mass index, height, and weight. Final models were refit using ordinary least squares with cluster‐robust standard errors to account for within‐person correlation. All continuous predictors and airway metrics were Z‐standardized. Color intensity denotes coefficient magnitude and sign (red = positive; blue = negative); blank cells indicate predictors not selected or not statistically significant. Abbreviations: CSA, cross‐sectional area; avg, average; cv, coefficient of variation.

The final multivariate linear regression results are visualized in Figure 4. Blank cells indicate predictors not selected by the model. Diagnostic assessments confirmed that the final regression models satisfied linear model assumptions. Variance‐inflation factors for all retained predictors were below the conventional threshold of 10 (typically 1–2), indicating low multicollinearity. Moreover, Normal Q–Q plots of standardized residuals showed no apparent departures from normality, while scatterplots of residuals versus fitted values exhibited an even spread, indicating homoscedasticity.

Male sex showed positive standardized coefficients across all mean airway metrics (Standardized *β* = 0.44–1.55) but was not retained in any CV models.

Open‐mouth status was associated with increased mean total airway length (Standardized *β* = 0.50) and volume (Standardized *β* = 0.25), and decreased mean retropalatal CSA (Standardized *β* = −0.46). It was also included in all CV models (Standardized *β* = 0.59–0.69) except for that of the epiglottic airway.

Symptomatic status was selected in three models, showing a positive association with mean total airway length (Standardized *β* = 0.32) and the CV of retropalatal CSA (Standardized *β* = 0.50), and a negative association with mean retropalatal CSA (Standardized *β* = −0.61).

Continuous anthropometric variables were included inconsistently across models. Age showed positive coefficients for epiglottic airway length (standardized *β* = 0.16), total airway length (standardized *β* = 0.34), and total airway volume (standardized *β* = 0.26). Neck circumference was retained only in the model for mean epiglottic airway volume (standardized *β* = 0.19). Height contributed solely to mean total airway length (standardized *β* = 0.47). BMI was not selected in any model. Weight contributed positive coefficients across all retained CV models (standardized *β* = 0.16–0.27) except for epiglottic airway and did not enter any mean models.

## 4 Discussion

The utilization of 4D dynamic MRI in this study revealed that the transition from closed‐ to open‐mouth breathing significantly alters both the geometry and the dynamic stability of the upper airway. The open‐mouth position was observed to significantly elongate the upper airway while narrowing the retropalatal cross‐sectional area.

This retropalatal narrowing has been attributed in previous studies to the posterior displacement of the soft palate driven by mandibular movement [24], and the accompanying airway elongation is consistent with previous cephalometric findings [25]. Our results confirmed the airway elongation by using 3D dynamic MRI under direct visualization and real time monitoring. Beyond these geometric changes, the dynamic analysis revealed significantly increased temporal variability across all airway measurements, as evidenced by higher CVs. This elevated temporal variability suggests that open‐mouth breathing may render the airway structure more susceptible to fluctuations during the respiratory cycle. Physiologically, this heightened variability may stem from the combined aerodynamic consequences of airway elongation and localized retropalatal narrowing.

First, the elongation of the pharyngeal tube may increase airflow resistance, consistent with Poiseuille's law, thereby amplifying the negative intraluminal pressure required to maintain ventilation. Furthermore, as airflow accelerates through the focal retropalatal bottleneck, it may generate increased negative pressure in accordance with the Bernoulli principle. The cumulative impact of these aerodynamic forces on the compliant pharyngeal soft tissues might destabilize the airway walls, potentially predisposing them to dynamic fluctuation, although airflow and intraluminal pressure were not directly measured in this study.

In contrast to significant retropalatal narrowing, the retroglossal CSA remained preserved in this study. This finding diverges from the multilevel collapse typically observed in OSA patients [26]. In these patients, the susceptibility to retroglossal collapse has been associated with compromised neuromuscular function such as snore‐induced neuropathy [27] and impaired muscle responsiveness [28]. This finding may suggest that intact neuromuscular reflexes in healthy individuals provide sufficient compensatory tone to maintain retroglossal dimensions despite mouth opening. Finally, the observation that epiglottic airway dimensions remained unchanged across mouth positions indicates that the mechanical impact of mouth opening exerts minimal influence on this caudal region.

In this cohort of adults free of the diagnosis of OSA, some of them reported sleep‐related breathing symptoms. Regardless of mouth position, these symptomatic participants exhibited significantly greater airway length and volume compared to asymptomatic controls. Multivariate regression also confirmed symptomatic status as an independent positive predictor of total airway length and volume. Crucially, this elongation parallels the structural remodeling typically observed in established obstructive sleep apnea patients [29, 30, 31]. This suggests that upper airway lengthening may represent an early morphological alteration that places these individuals on a trajectory toward pathology. Similar to the geometric disadvantage induced by mouth opening, this intrinsic elongation likely increases aerodynamic resistance, thereby precipitating symptoms such as snoring even before overt apnea develops. Specific to the open‐mouth position, the symptomatic group displayed significantly narrower retropalatal cross‐sectional areas and elevated coefficients of variation for this specific region. This anatomical profile is also observed in patients with obstructive sleep apnea [29, 30, 31], where retropalatal narrowing is typically attributed to soft tissue hypertrophy and parapharyngeal fat deposition which encroach upon the airway lumen [9, 32]. This suggests that retropalatal narrowing may also represent an early morphological change in this population. Physiologically, the observed regional dynamic instability likely stems from this focal bottleneck which induces a local negative intraluminal pressure. These data may provide a morphological basis for interventions targeting this level such as uvulopalatopharyngoplasty [33, 34] and support clinical recommendations favoring nasal breathing [4, 26, 35].

Regarding sexual dimorphism, male participants generally exhibited larger airway dimensions than females, a finding consistent with the broader craniofacial bony structures typically observed in males [36]. A notable exception in the descriptive analysis was the retropalatal CSA, which appeared comparable between sexes. However, multivariate regression clarified this apparent discrepancy by identifying male sex as a significant positive predictor for retropalatal CSA after adjusting for confounders. This suggests that the underlying anatomical size advantage in males was masked in the univariate analysis by the higher prevalence of symptomatic individuals within the male cohort. Since symptomatic status is associated with retropalatal narrowing, its disproportionate presence among males likely offset their baseline anatomical advantage. In terms of dynamics, the similar CVs across measurements suggest that males and females exhibit comparable levels of airway movement during tidal breathing. However, given the relatively wide confidence intervals associated with these estimates, any conclusions regarding sex‐specific dynamic variability should be drawn with caution.

Moreover, multivariate analysis elucidated the impact of anthropometric variables on airway morphology. Age was identified as a significant positive predictor of airway length. This observation is consistent with established age‐related anatomical changes described in the literature [37]. Specifically, this lengthening is typically attributed to the progressive caudal descent of laryngeal structures associated with aging [38]. Regarding body habitus, absolute body weight was positively associated with the CVs across most airway dimensions. This finding might support that the total mass of soft tissue imposes a direct mechanical load on the pharyngeal structures [39, 40].

The comprehensive dynamic characterization presented in this study was underpinned by the active‐learning nnU‐Net framework. Four successive generations of models were developed to address the challenge of segmenting high‐volume 4D‐MRI data. The stability of the model progressively improved, as evidenced by the fourth‐generation model's substantial decrease in segmentation entropy, reduction in low‐Dice outliers, and achievement of a higher minimum dice coefficient in both the hold‐out test set and validation datasets. This technical robustness confirms that the active learning pipeline effectively minimizes manual annotation burden while maintaining the high segmentation accuracy necessary for the precise quantification of airway morphology and dynamics.

## 5 Limitations

First, the TWIST sequence's near‐real‐time acquisition (∼1 fps at 2.5 mm voxels) sacrifices spatial resolution and is still susceptible to motion and breathing artifacts during scanning. The short 20‐s window also spans only a few breaths and cannot capture sporadic collapse events; therefore, future acquisition protocols will employ longer imaging windows to capture a greater number of respiratory cycles. Second, the nnU‐Net was trained exclusively on healthy Taiwanese adults using a single TWIST protocol, limiting its generalizability to other scanners, sequences, or OSA patients. Third, the use of a gauze‐wrapped 10‐mL syringe inherently introduces inter‐individual variability in the degree of jaw opening due to anatomical differences. However, post hoc analysis demonstrated that this variability was not significantly correlated with the airway metrics, suggesting that the variation in jaw position in this study did not significantly confound the morphological findings. Fourth, the modest sample size and strong predictor collinearity may predispose the multivariate models to overfitting, and unmeasured factors such as lung volume and respiratory effort may have confounded our findings.

## 6 Conclusions

This study demonstrates that integrating four‐dimensional dynamic MRI with an active learning nnU‐Net enables the automated and comprehensive assessment of upper airway morphology with reduced requirements for manual labeling. Beyond static metrics, this approach captures dynamic behaviors and suggests that open‐mouth breathing may compromise airway stability, accompanied by specific geometric alterations such as elongation of the upper airway. The observation that OSA‐free symptomatic individuals exhibit similar morphological traits suggests that these anatomical deviations may serve as early indicators of functional vulnerability. Ultimately, this scalable and non‐invasive modality provides deeper insights into respiratory physiology and holds promise for enhancing early risk stratification and informing personalized therapeutic interventions.

## Funding

This work was supported by the National Science and Technology Council, R.O.C. Taiwan (113‐2314‐B‐002‐016, 114‐2628‐E‐002‐019‐MY3), the National Health Research Institutes, R.O.C. Taiwan (NHRI‐EX115‐11205EC), the Yushan Fellow Program (NTU‐113V1015‐4, NTU‐114V1015‐5), and the Higher Education Sprout Program (NTU‐115L7770, NTU‐115L7848, NTU‐114L900703).

## Supporting information


**Data S1:** Supporting Information.

## Data Availability

The MRI datasets analyzed during the current study contain potentially identifying personal information and, in accordance with institutional policies and participant consent, cannot be shared publicly. De‐identified data may be made available from the corresponding author upon reasonable request and subject to appropriate data use agreements. The trained weights of the airway segmentation model will be released online upon publication, together with minimal inference scripts.
