# Supplementary material for: Analysis of Upper Airway Morphology Using Four‐Dimensional Dynamic MRI With Active Deep Learning‐Based Automatic Segmentation
Source: J Magn Reson Imaging. 2026 Jan 28;63(5):1404–17. doi: 10.1002/jmri.70237 (PMC13066510; doi:10.1002/jmri.70237)
Supplement: Supplementary file 1 — Data S1: Supporting Information. [file JMRI-63-1404-s001.docx]

**Supplementary Table S1** Comparison of upper airway metrics between closed- and open-mouth statuses (n = 84).

| **Feature** | | **Closed-mouth** | **Open-mouth** | **Δ(open − closed) (95% CI)** | **p-value** |
| --- | --- | --- | --- | --- | --- |
| **Twenty-Second Means** | | | | | |
| Length (cm) | Epiglottic airway | 1.28 ± 0.24 (1.13, 1.40) | 1.29 ± 0.23 (1.18, 1.43) | 0.02 (-0.02, 0.05) | 0.298 |
| CSA (cm^2^) | Average | 3.03 ± 0.53 (2.66, 3.30) | 3.02 ± 0.57 (2.62, 3.36) | -0.01 (-0.11, 0.08) | 0.821 |
|  | Retroglossal | 3.62 ± 0.85 (3.21, 4.06) | 3.56 ± 1.05 (2.91, 3.89) | -0.06 (-0.25, 0.13) | 0.524 |
| Volume (cm^3^) | Epiglottic airway | 2.86 ± 1.13 (2.16, 3.55) | 2.88 ± 1.18 (2.23, 3.41) | 0.18(-1.56, 1.92) | 0.839 |

This table includes only prespecified contrasts that were not statistically significant (two-sided p ≥ 0.05). Within-status values are presented as mean ± SD (25th, 75th percentiles). Differences are defined as open minus closed, and are reported as mean differences for parametric outcomes. Ninety-five percent confidence intervals are t-based for parametric outcomes. CSA denotes Cross-sectional area.

**Supplementary Table S2**. Upper airway metrics under closed- and open-mouth statuses stratified by sex (male, n = 28; female, n = 56), prespecified nonsignificant contrasts.

S2a. Closed-mouth

| **Feature** | | **Male(n=28)** | **Female(n=56)** | **Δ(Male-Female) (95% CI)** | **p-value** |
| --- | --- | --- | --- | --- | --- |
| **Twenty-Second Means** | | | | | |
| CSA (cm^2^) | Retropalatal | 1.94 ± 0.71 (1.47, 2.28) | 1.74 ± 0.68 (1.32, 2.13) | 0.2 (-0.13, 0.52) | 0.225 |
| **Twenty-Second Coefficients of Variation** | | | | | |
| CSA | Average | 0.03 ± 0.02 (0.02, 0.03) | 0.02 ± 0.01 (0.01, 0.03) | 0 (0, 0.01) | 0.317 |
|  | Retropalatal | 0.08 ± 0.06 (0.04, 0.09) | 0.06 ± 0.03 (0.03, 0.08) | 0.01 (-0.01, 0.04) | 0.170 |
|  | Retroglossal | 0.04 ± 0.03 (0.03, 0.06) | 0.04 ± 0.02 (0.02, 0.04) | 0.01 (0, 0.02) | 0.290 |
| Volume | Total airway | 0.03 ± 0.02 (0.02, 0.03) | 0.02 ± 0.01 (0.01, 0.03) | 0 (0, 0.01) | 0.317 |
|  | Epiglottic airway | 0.06 ± 0.06 (0.03, 0.06) | 0.06 ± 0.06 (0.03, 0.07) | 0 (-0.01, 0.02) | 0.673 |

S2b. Open-mouth

| **Feature** | | **Male(n=28)** | **Female(n=56)** | **Δ(Male-Female) (95% CI)** | **p-value** |
| --- | --- | --- | --- | --- | --- |
| **Twenty-Second Means** | | | | | |
| CSA (cm^2^) | Retropalatal | 1.64 ± 0.66 (1.09, 2.15) | 1.44 ± 0.68 (1.04, 1.67) | 0.2 (-0.11, 0.51) | 0.206 |
| **Twenty-Second Coefficients of Variation** | | | | | |
| CSA | Average | 0.05 ± 0.03 (0.02, 0.06) | 0.04 ± 0.03 (0.02, 0.05) | 0.01 (-0.01, 0.02) | 0.189 |
|  | Retropalatal | 0.14 ± 0.13 (0.05, 0.18) | 0.11 ± 0.11 (0.05, 0.12) | 0.01 (-0.02, 0.1) | 0.145 |
|  | Retroglossal | 0.08 ± 0.06 (0.03, 0.09) | 0.06 ± 0.03 (0.03, 0.08) | 0.01 (-0.02, 0.03) | 0.303 |
| Volume | Total airway | 0.05 ± 0.03 (0.02, 0.06) | 0.04 ± 0.03 (0.02, 0.05) | 0.01 (-0.01, 0.02) | 0.189 |
|  | Epiglottic airway | 0.08 ± 0.07 (0.04, 0.08) | 0.08 ± 0.07 (0.04, 0.09) | -0.01 (-0.02, 0.01) | 0.462 |

This table includes only prespecified contrasts that were not statistically significant (two-sided p ≥ 0.05). S2a Closed-mouth status; S2b Open-mouth status. Within-group values are presented as mean ± SD (25th, 75th percentiles). Differences are defined as male minus female and are reported as mean differences for parametric outcomes and median differences for non-parametric outcomes. Ninety-five percent confidence intervals are t-based for parametric outcomes and percentile-bootstrap for non-parametric outcomes. CSA denotes cross-sectional area.

**Supplementary Table S3** Upper airway metrics under closed‐ and open‐mouth statuses, stratified by symptom status (asymptomatic, n = 51; symptomatic, n = 33), prespecified nonsignificant contrasts.

S3a Closed-mouth status

| **Feature** | | **Asymptomatic(n=51)** | **Symptomatic (n=33)** | **Δ(Symptomatic-Asymptomatic) (95% CI)** | **p-value** |
| --- | --- | --- | --- | --- | --- |
| **Twenty-Second Means** | | | | | |
| CSA (cm^2^) | Average | 3.04 ± 0.57 (2.66, 3.37) | 3.00 ± 0.47 (2.63, 3.24) | -0.04 (-0.27, 0.19) | 0.721 |
|  | Retropalatal | 1.91 ± 0.67 (1.47, 2.22) | 1.63 ± 0.70 (1.03, 2.00) | -0.29 (-0.59, 0.02) | 0.067 |
|  | Retroglossal | 3.56 ± 0.89 (3.20, 4.01) | 3.72 ± 0.78 (3.27, 4.44) | 0.16 (-0.21, 0.53) | 0.390 |
| Volume (cm^3^) | Epiglottic airway | 2.67 ± 1.05 (1.84, 3.33) | 3.15 ± 1.20 (2.40, 4.11) | 0.48 (-0.04, 1) | 0.068 |
| **Twenty-Second Coefficients of Variation** | | | | | |
| CSA | Average | 0.02 ± 0.01 (0.01, 0.03) | 0.03 ± 0.02 (0.02, 0.04) | 0 (-0.01, 0.01) | 0.175 |
|  | Retroglossal | 0.04 ± 0.02 (0.02, 0.04) | 0.05 ± 0.03 (0.03, 0.06) | 0 (-0.01, 0.02) | 0.314 |
| Volume | Total airway | 0.02 ± 0.01 (0.01, 0.03) | 0.03 ± 0.02 (0.02, 0.04) | 0 (0, 0.01) | 0.175 |
|  | Epiglottic airway | 0.06 ± 0.05 (0.03, 0.07) | 0.06 ± 0.06 (0.03, 0.06) | 0 (-0.01, 0.02) | 0.869 |

S3b Open-mouth status

| **Feature** | | **Asymptomatic(n=51)** | **Symptomatic (n=33)** | **Δ(Symptomatic-Asymptomatic) (95% CI)** | **p-value** |
| --- | --- | --- | --- | --- | --- |
| **Twenty-Second Means** | | | | | |
| CSA (cm^2^) | Average | 2.97 ± 0.55 (2.49, 3.27) | 3.09 ± 0.61 (2.66, 3.53) | 0.12 (-0.14, 0.38) | 0.374 |
| **Twenty-Second Coefficients of Variation** | | | | | |
| CSA | Average | 0.04 ± 0.03 (0.02, 0.05) | 0.05 ± 0.03 (0.02, 0.06) | 0.01 (-0.01, 0.02) | 0.172 |
|  | Retroglossal | 0.06 ± 0.03 (0.03, 0.07) | 0.08 ± 0.05 (0.04, 0.09) | 0.02 (-0.01, 0.03) | 0.126 |
| Volume | Total airway | 0.04 ± 0.03 (0.02, 0.05) | 0.05 ± 0.03 (0.02, 0.06) | 0.01 (-0.01, 0.02) | 0.172 |
|  | Epiglottic airway | 0.08 ± 0.07 (0.04, 0.09) | 0.08 ± 0.07 (0.04, 0.09) | -0.01 (-0.02, 0.02) | 0.783 |

This table includes only prespecified contrasts that were not statistically significant (two-sided p ≥ 0.05). S3a Closed-mouth status; S3b Open-mouth status. Within-group values are presented as mean ± SD (25th, 75th percentiles). Differences are defined as symptomatic minus asymptomatic and are reported as mean differences for parametric outcomes and median differences for non-parametric outcomes. Ninety-five percent confidence intervals are t-based for parametric outcomes and percentile-bootstrap for non-parametric outcomes. CSA denotes cross-sectional area.

**Supplementary Material S4** Assessment of Mouth Opening Variability and its Impact on Airway Metrics

To directly evaluate whether the variation in mouth opening influenced the airway metrics, we retrospectively quantified the extent of mouth opening on the midsagittal MR images. The measurement procedure was as follows: 1) The palatal plane was identified as the reference baseline. 2) The perpendicular distance from this plane to the most inferior point of the mandibular symphysis was measured in both the closed mouth scan, denoted as $d_{close}$ and the open mouth scan, denoted as $d_{open}$. 3) The variation in mouth opening was defined as: $\Delta d = d_{open}- d_{close}$.

Correlation analyses were performed to test whether $\Delta d$ had a measurable impact on the primary open mouth airway outcomes. For metrics that followed a normal distribution, such as airway length, cross sectional area (CSA), and volume, Pearson correlation was implemented. For measures of dynamic stability, which are the 20 second coefficient of variation (CV), Spearman rank correlation was utilized.

Across the cohort of 84 participants, the mean difference between the open mouth and closed mouth distances $\Delta d$ was 16.77±3.73 mm. The detailed correlation coefficients, p values, and confidence intervals, along with a schematic diagram illustrating the measurement of $\Delta d$, are provided below. The correlation analysis demonstrated that $\Delta d$ was not significantly correlated with any of the primary open mouth airway outcomes. These results suggest that, under the syringe-based standardization protocol used in this study, the inter individual differences in the extent of mouth opening do not represent a major linear determinant of airway morphology or dynamic stability.

| 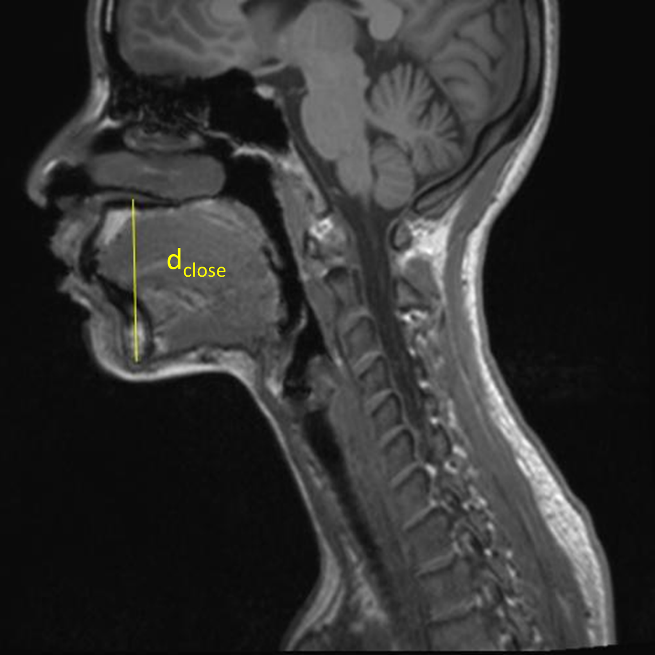 | 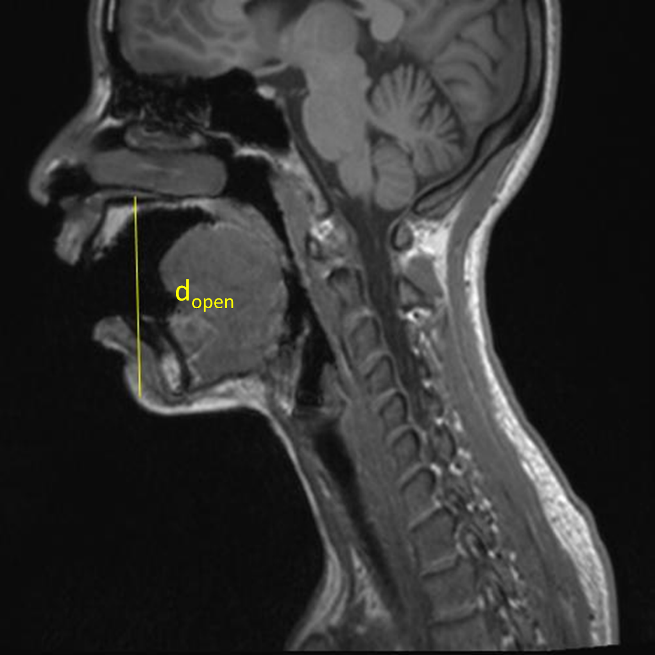 |
| --- | --- |

Figure S1. Schematic Illustration of Mouth Opening Measurement.

Representative midsagittal MR images demonstrating the quantification of mouth opening. The left panel shows the closed mouth position $d_{close}$, and the right panel shows the open mouth position $d_{open}$ achieved using the syringe bite block. The yellow vertical lines represent the perpendicular distance measured from the palatal plane to the inferior point of the mandibular symphysis.

| **Feature** | | **Pearson r** | **p-value** | **95% CI** |
| --- | --- | --- | --- | --- |
| **Twenty-Second Means** | | | | |
| Length (cm) | Total airway | 0.008 | 0.946 | [-0.207, 0.222] |
|  | Epiglottic airway | -0.004 | 0.971 | [-0.218, 0.210] |
| CSA (cm2) | Average | 0.117 | 0.288 | [-0.100, 0.323] |
|  | Retropalatal | 0.004 | 0.968 | [-0.210, 0.219] |
|  | Retroglossal | 0.038 | 0.729 | [-0.177, 0.251] |
| Volume (cm3) | Total airway | 0.089 | 0.422 | [-0.128, 0.298] |
|  | Epiglottic airway | 0.048 | 0.667 | [-0.168, 0.259] |

Table S4a. Pearson Correlation Analysis for Mean Airway Metrics

Pearson correlation analysis between the extent of mouth opening $\Delta d$ and the twenty second mean airway metrics across the cohort of 84 participants. The table presents the Pearson correlation coefficient r, the associated p-value, and the 95% confidence interval (CI) for each morphological measurement.

| **Feature** | | **Spearman ρ** | **p-value** | **95% CI** |
| --- | --- | --- | --- | --- |
| **Twenty-Second Coefficients of Variation** | | | | |
| CSA (cm2) | Average | -0.073 | 0.507 | [-0.283, 0.143] |
|  | Retropalatal | -0.101 | 0.358 | [-0.309, 0.115] |
|  | Retroglossal | 0.017 | 0.881 | [-0.199, 0.23] |
| Volume (cm3) | Total airway | -0.073 | 0.507 | [-0.283, 0.143] |
|  | Epiglottic airway | -0.049 | 0.656 | [-0.167, 0.261] |

Table S4b. Spearman Rank Correlation Analysis for Airway Variability

Spearman rank correlation analysis between the extent of mouth opening$\Delta d$ and the twenty second coefficients of variation (CV) across the cohort of 84 participants. The table presents the Spearman ρ, the associated p-value, and the 95% confidence interval (CI) for the dynamic variability of each airway metric.
